# Novel hollow α-Fe_2_O_3_ nanofibers via electrospinning for dye adsorption

**DOI:** 10.1186/s11671-015-0874-7

**Published:** 2015-04-14

**Authors:** Qiang Gao, Jun Luo, Xingyue Wang, Chunxia Gao, Mingqiao Ge

**Affiliations:** Key Laboratory of Science and Technology of Eco-Textiles, Ministry of Education, Jiangnan University, 1800 Lihu Road, Wuxi, 214122 China; State Key Laboratory of Molecular Engineering of Polymers, Department of Macromolecular Science and Laboratory of Advanced Materials, Fudan University, 220 Handan Road, Shanghai, 200438 China; Institute of Orthopaedics, The First Affiliated Hospital, Soochow University, 708 Renmin Road, Suzhou, 215006 China

**Keywords:** Hollow nanofiber, Electrospinning, α-Fe_2_O_3_, Magnetic

## Abstract

Nanomaterials such as iron oxides and ferrites have been intensively investigated for water treatment and environmental remediation applications. In this work, hollow α-Fe_2_O_3_ nanofibers made of rice-like nanorods were successfully synthesized via a simple hydrothermal reaction on polyvinyl alcohol (PVA) nanofiber template followed by calcination. The crystallographic structure and the morphology of the as-prepared α-Fe_2_O_3_ nanofibers were characterized by X-ray diffraction, energy dispersive X-ray spectrometer, and scanning electron microscope. Batch adsorption experiments were conducted, and ultraviolet-visible spectra were recorded before and after the adsorption to investigate the dye adsorption performance. The results showed that hollow α-Fe_2_O_3_ fiber assembles exhibited good magnetic responsive performance, as well as efficient adsorption for methyl orange in water. This work provided a versatile strategy for further design and development of functional nanofiber-nanoparticle composites towards various applications.

## Background

In recent decades, magnetic iron oxide has developed into a kind of nanomaterial with the property of magnetic targeting [1,2]. α-Fe_2_O_3_ has attracted considerable attention due to its widely applications, such as catalysis [3-5], batteries [6,7], and gas sensors [8,9]. Recently, much effort has been devoted to the design and controllable synthesis of one-dimensional (1D) nanostructure α-Fe_2_O_3_ due to the novel properties of nanoscale materials. Liu et al*.* synthesized α-Fe_2_O_3_ nanotubes by a templating method [10]. Jiang’s group and Gou’s research group have fabricated α-Fe_2_O_3_ nanofibers via electrospinning, respectively [11,12].

Electrospinning is a simple method for producing nanofibers and nonwovens for various applications [13-16]. Electrospinning is advantageous to fabricate not only solid nanofibers but also hollow nanofibers. The main strategies adopted for hollow nanofiber synthesis via electrospinning are as follows: (1) *Coaxial electrospinning* involves the use of two coaxial capillaries in a spinneret containing different solutions to generate core-shell composite fibers that results in hollow fibers via removal of core fibers by extraction or calcination at high temperature [17,18]. (2) *Single nozzle co-electrospinning*: this process involves two immiscible polymers dissolved in solvent that results in phase separation during electrospinning owing to the intrinsic polymer properties, yielding core-shell composite fibers or hollow fibers after suitable core removal [19,20]. However, inorganic hollow fiber with hierarchical structure is hard to be achieved via the two mentioned methods.

Nanoscale α-Fe_2_O_3_ has been intensively investigated for water treatment and environmental remediation applications [21-23]. Song et al. reported that flowerlike α-Fe_2_O_3_ nanoparticles can remove the heavy metal ions from the waste water [21]. Grätzel et al. employed nanostructured α-Fe_2_O_3_ films for azo-dye adsorption [22]. Yu et al. investigated the methyl orange degradation performance by using α-Fe_2_O_3_ nanocrystals [23]. To the best of our knowledge, there are no reports in the literature dealing with interconnected α-Fe_2_O_3_ hollow fibers for dye adsorption in the waste water. In this work, we have synthesized a interconnected 1D hollow structure of α-Fe_2_O_3_ nanofibers made of rice-like nanorods by annealing electrospun polyvinyl alcohol (PVA)-Fe_3_O_4_ composite fibers and investigated its potential applications in removal of noxious dye from wastewater.

## Methods

### Materials

99.9% hydrolyzed PVA samples (DP = 3,200) were provided by Kuraray Co. Ltd., Tokyo, Japan. Acetic acid, FeCl_3_·6H_2_O, FeCl_2_·4H_2_O, and sodium hydroxide were purchased from Wako, Osaka, Japan. All the reagents were used as received without further purification.

### Preparation of PVA nanofibers

PVA solutions were prepared by dissolving PVA in acetic acid aqueous solution at 90°C with constant stirring for at least 4 h. The electrospinning setup (Kato Tech, Kyoto, Japan) used in this study consists of a syringe with a flat-end metal needle (1.20-mm inner diameter, NN-1838 N, Terumo, Tokyo, Japan), a syringe pump for controlled the feeding rate, a grounded cylindrical stainless steel mandrel, and a high-voltage DC power supply. In a typical electrospinning process, PVA solution was transferred into a syringe and delivered to the tip of the syringe needle by the syringe pump at a constant feed rate (1.0 ml/h). A 12-kV positive voltage was applied to the PVA solution via the stainless steel syringe needle. The subsequently ejected polymer fiber was collected on the rotating cylindrical stainless steel mandrel, which was rotated during the electrospinning process (150 rpm). The distance between the tip of the needle and the surface of the mandrel was about 14 cm. The PVA nanofibers were vacuumed at room temperature for 24 h and thermal-treated at 180°C for 5 min.

### Preparation of hollow α-Fe_2_O_3_ nanofibers

FeCl_3_·6H_2_O and FeCl_2_·4H_2_O were dissolved in 8 ml of distilled water, and the aqueous solution was degassed by N_2_. The PVA nanofiber mat (0.5 mg) was immersed within the degassed aqueous solution of ferrous and ferric ions and stood for 1 h. After 1 h, NaOH aqueous solution (0.5 ml) was added slowly and the mixture was heated to 70°C for 60 min. After cooling to room temperature, the PVA-Fe_3_O_4_ composite mat was washed with water and dried *in vacuo* for 12 h.

**Figure 1:**
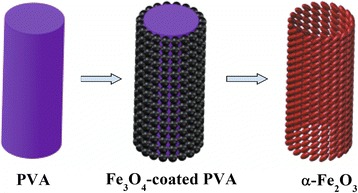
Schematic synthetic strategy for hollow α-Fe_2_O_3_ nanofibers.

As shown in Figure 1, the PVA-Fe_3_O_4_ composite mat was annealed in a tube furnace (GSL-1600X-80, Kejing, Hefei, China) at 600°C for 4 h under air to remove the PVA template and form hollow α-Fe_2_O_3_ nanofibers.

### Characterization

Fiber morphology of the electrospun fibers was characterized using scanning electron microscopy (SEM) (SU1510, Hitachi Co. Ltd., Tokyo, Japan) and field-emission scanning electron microscopy (FE-SEM) (JSM-6700 F, JEOL, Akishima-shi, Japan) with an energy-dispersive X-ray spectrometer (EDS). Differential scanning calorimeter (DSC) (Q200, TA Instruments Inc., New Castle, USA) was used to characterize the thermal properties of the electrospun PVA mats. A piece of PVA mat (2 to 5 mg) was placed in an aluminum sample pan and heated from 30°C to 350°C at 10°C/min under N_2_. Weight loss behavior was tested by thermogravimetric (TG) analysis (SDT Q600, TA Instruments Inc., New Castle, USA) (air, 10°C/min). The chemical structure of nanofibers was conducted with a Fourier transform infrared (FT-IR) reflection spectroscopy (NICOLET, Thermo Fisher Scientific, Waltham, USA), and a X-ray powder diffractometer (XRD) (D8 Advance, Bruker, Karlsruhe, Germany) operated in the reflection mode with Cu-Kα radiation in the 2*θ* range of 10° to 80° with a rate of 4°/min. Batch adsorption experiments were conducted and recorded by ultraviolet-visible spectra (U-3010, Hitachi Co. Ltd., Tokyo, Japan).

## Results and discussions

### Deposition of Fe_3_O_4_ nanoparticles on PVA nanofibers

**Figure 2:**
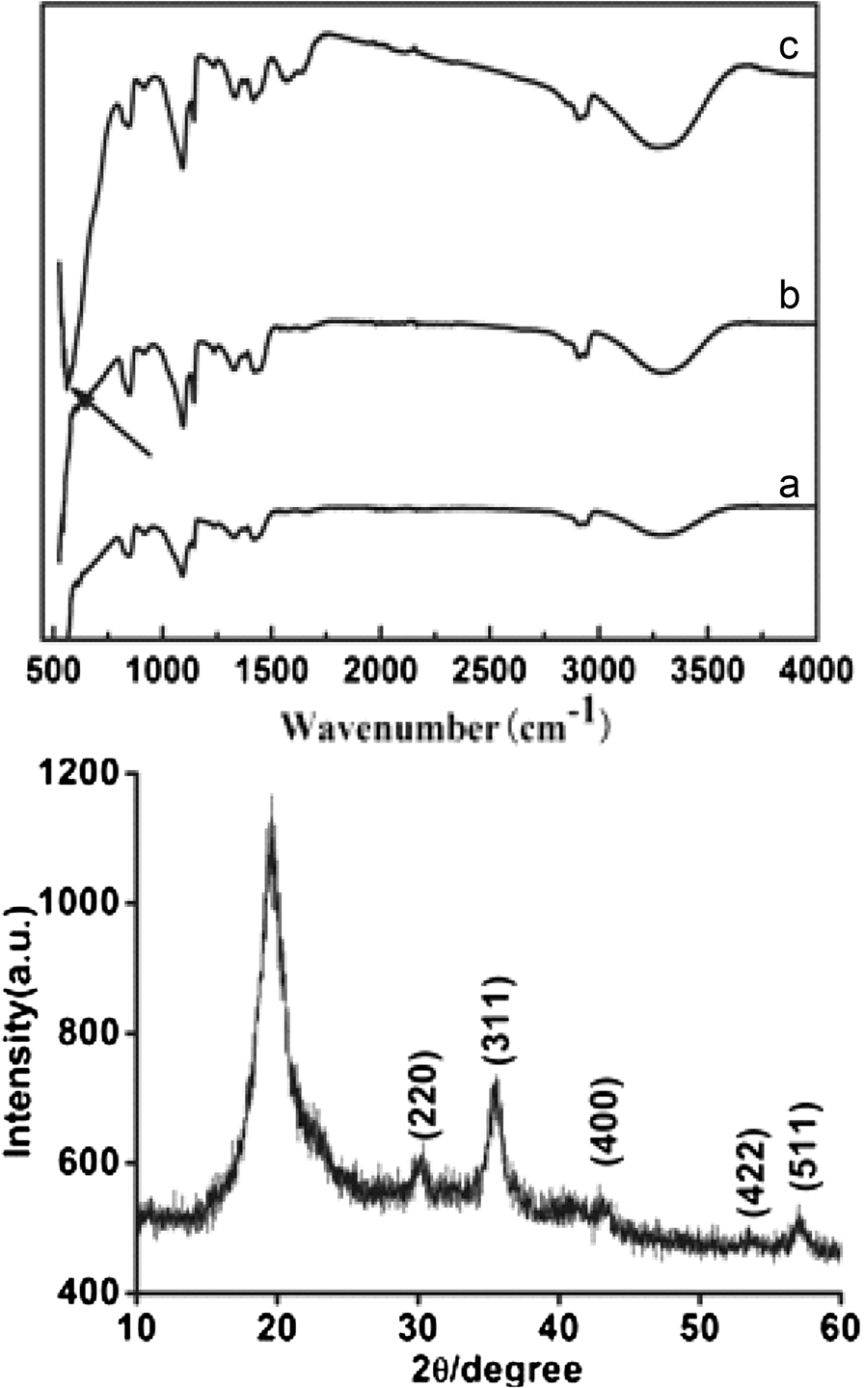
Infrared spectra of PVA nanofibers. **(a)** As-spun; **(b)** after thermal treatment; **(c)** coated with magnetite layer and its XRD pattern.

The formation of Fe_3_O_4_ on the PVA nanofibers can be monitored by the FT-IR spectra and XRD pattern. Figure 2 shows FT-IR spectra of as-spun PVA nanofibers, PVA nanofibers after heat treatment, and PVA nanofibers deposited with magnetite layer together with its XRD pattern. A broad characteristic peak at 3,300 cm^−1^ attributed to the -OH stretching vibration of PVA [13]. Compared to Figure 2a, no new absorbance band was observed in the spectrum of PVA nanofibers after heat treatment (Figure 2b), indicating only the physical changes that occur in the heat treatment process. In Figure 2c, the absorption peak at 567 cm^−1^ as the characteristic peak of Fe-O bonds in Fe_3_O_4_ was observed [24], indicating that successful hydrothermal synthesis of Fe_3_O_4_ on the surface of PVA nanofibers. Moreover, the formation of Fe_3_O_4_ was further confirmed by the characteristic peaks observed in the XRD pattern. As shown in Figure 2, the XRD pattern of PVA nanofibers deposited with magnetite layer showed five diffraction peaks at 2*θ* of 30.2°, 35.6°, 43.3°, 53.5°, and 57.2°. These peak positions agree with (220), (311), (400), (422), and (511) crystallographic planes of the spinel phase of Fe_3_O_4_ [25].

**Figure 3:**
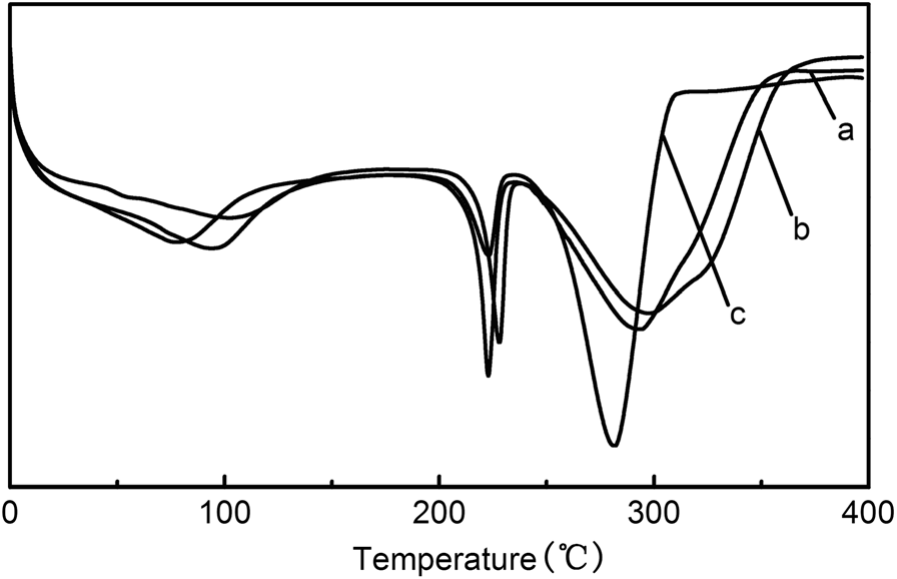
DSC curves of PVA nanofibers **(a)** as-spun, **(b)** after thermal treatment, and **(c)** coated with Fe_3_O_4_ magnetic nanoparticles.

Figure 3 shows the DSC curves of as-spun PVA nanofibers, PVA nanofibers after heat treatment, and PVA nanofibers coated with Fe_3_O_4_ nanoparticles. The enthalpy of 100% crystalline PVA is 138.6 J/g [26]. From Table 1, the crystallinity of as-spun PVA nanofibers is 51.4%. After heat treatment at 180°C for 5 min, the crystallinity of PVA nanofibers increased to 56.7%, which is due to the removal of H_2_O in the PVA matrix, so that the hydrogen bonding between PVA macromolecules is enhanced, thereby promote the crystallization. After the hydrothermal reaction for the deposition of Fe_3_O_4_ nanoparticles, the crystallinity of PVA-Fe_3_O_4_ composite decreased from 56.7% to 31.4%. Meanwhile, the melting point slightly decreased from 223°C to 222°C. During the process of hydrothermal synthesis, some water molecules squeezed into the PVA molecular chains, weakened the hydrogen bonding of -OH groups.

**Table 1:** **The melting point and enthalpy of PVA nanofibers**

**PVA nanofibers**	**Tm (°C)**	**Δ** ***H*** **(J/g)**
As-spun fibers	227	71.3
After thermal treatment	223	78.5
PVA-Fe_3_O_4_ composite fibers	222	43.5

### Hollow α-Fe_2_O_3_ nanofibers

**Figure 4:**
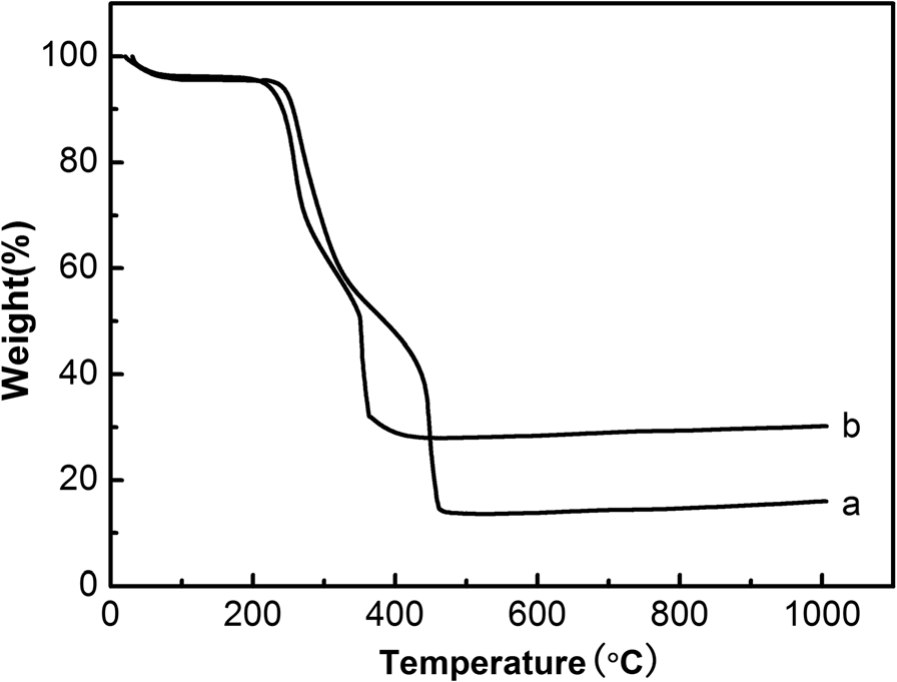
Thermogravimetric analysis curves of PVA nanofibers **(a)** after heat treatment and **(b)** coated with Fe_3_O_4_ magnetic nanoparticles.

Figure 4 shows the TG curves of PVA nanofibers before and after the hydrothermal reaction. After the heat treatment, PVA nanofibers reveal good thermal stability below 229°C. Weight loss occurs in the region of 240°C to 350°C, probably because of dehydration of hydroxyl groups within the skeleton PVA molecules. Weight loss between 350°C to 470°C was mainly induced by the decomposition of C-C skeleton. Over 470°C, the weight of PVA basically unchanged which implies that PVA has been completely removed via calcination. In Figure 3b, PVA-Fe_3_O_4_ composite fibers showed a difference of 17 wt% in resultant weight over 500°C compared to PVA nanofibers which generated nonwoven fabrics of iron oxide.

**Figure 5:**
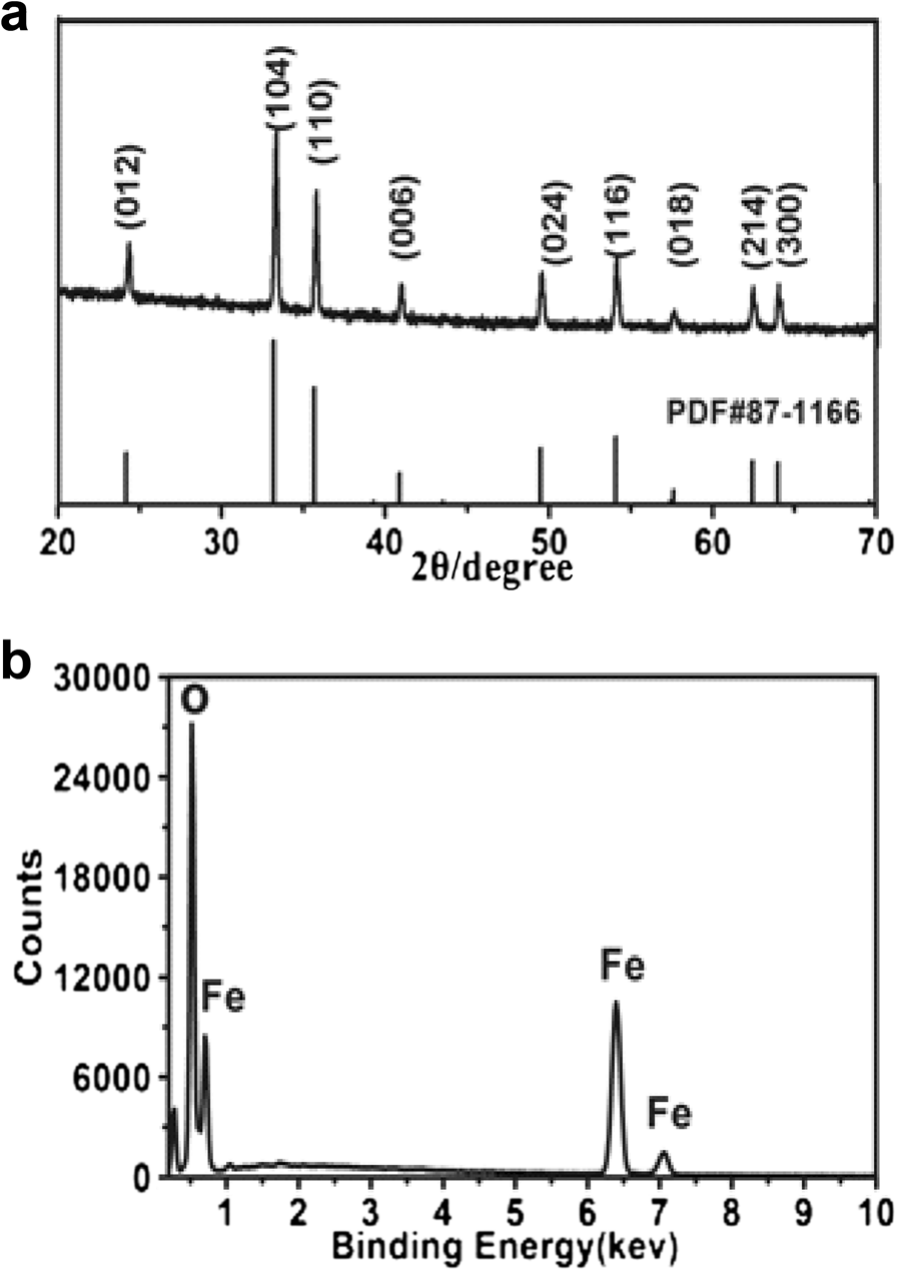
XRD **(a)** and EDS **(b)** patterns of the calcined product prepared at 600°C for 4 h under air.

Figure 5a shows the XRD pattern of the calcined product prepared at 600°C for 4 h under air. The diffraction peaks at 2*θ* of 24.1°, 33.2°, 35.6°, 40.8°, 49.4°, 54.1°, 57.6°, 62.4°, and 64.0° correspond to (012), (104), (110), (006), (024), (116), (018), (214), and (300) crystallographic planes of hematite structure of α-Fe_2_O_3_ by comparison with JCPDS card files number 87-1166 (*a* = 5.038 Å, *c* = 13.756 Å) [27]. The strong and sharp diffraction peaks indicate good crystallinity of the calcined product. No characteristic peaks from impurities are detected. The formation of Fe_2_O_3_ was further confirmed by EDS pattern (Figure 5b) of the calcined product. Fe and O peaks can be clearly seen.

**Figure 6:**
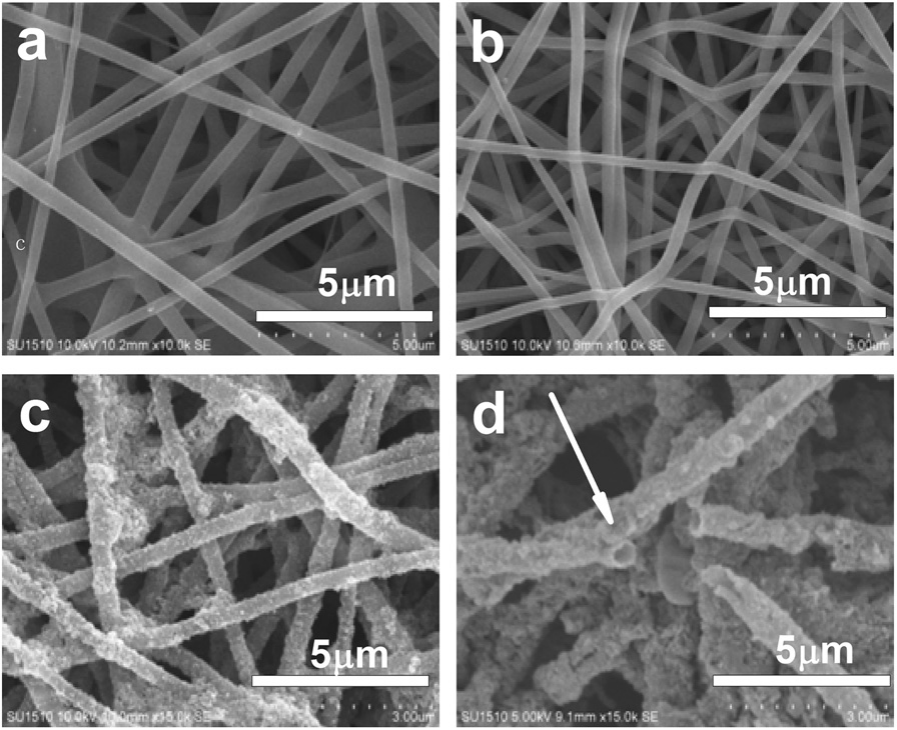
SEM images of PVA nanofibers. **(a)** As-spun; **(b)** after thermal treatment; **(c)** coated with Fe_3_O_4_ magnetic nanoparticles; **(d)** after calcination at 600°C for 4 h under air.

As shown in Figure 6a, the as-spun PVA nanofibers with an average diameter of 311 ± 66 nm are straight and reveal uniform and smooth surfaces. After heat treatment, PVA nanofibers are bent and the diameter of the fiber becomes slightly thicker, increased to 328 ± 55 nm, which may be due to the annealing during the heat treatment process. During the deposition process of magnetite, the color of nanofiber mat was changed from white to pale brown. The SEM image revealed a uniform deposition of Fe_3_O_4_ layer onto the surface of PVA nanofibers and no formation of large magnetite particles (Figure 6c). The average diameter of Fe_3_O_4_ nanoparticle-coated PVA nanofibers was 380 ± 53 nm; thus, the layer thickness of Fe_3_O_4_ nanoparticles deposited on PVA nanofibers was about 26 nm. The uniform deposition of Fe_3_O_4_ layer is mainly attributed to the complexation of iron ions or Fe_3_O_4_ nanoparticles with the hydroxyl groups in PVA. After calcination at 600°C for 4 h under air, intact nanofibrous morphology remained, but hollow structure was approached (as indicated by an arrow in Figure 6d). With the calcination temperature increased gradually, PVA nanofibers decomposed, interconnected Fe_3_O_4_ nanoparticles maintained the fibrous morphology, and eventually transformed to α-Fe_2_O_3_ nanofibers with hollow structure.

The geometrical parameters of PVA nanofibers, such as the average diameter of nanofibers, the porosity, and the thickness of fiber assembles, would significantly influence the resultant morphology of α-Fe_2_O_3_ nanofibers. Furthermore, the hydrothermal reaction conditions, such as temperature and ion concentration, should have similar effects. Here, the effect of ion concentration on the resultant morphology of α-Fe_2_O_3_ was investigated. Other parameters will be reported in the following full paper.

**Figure 7:**
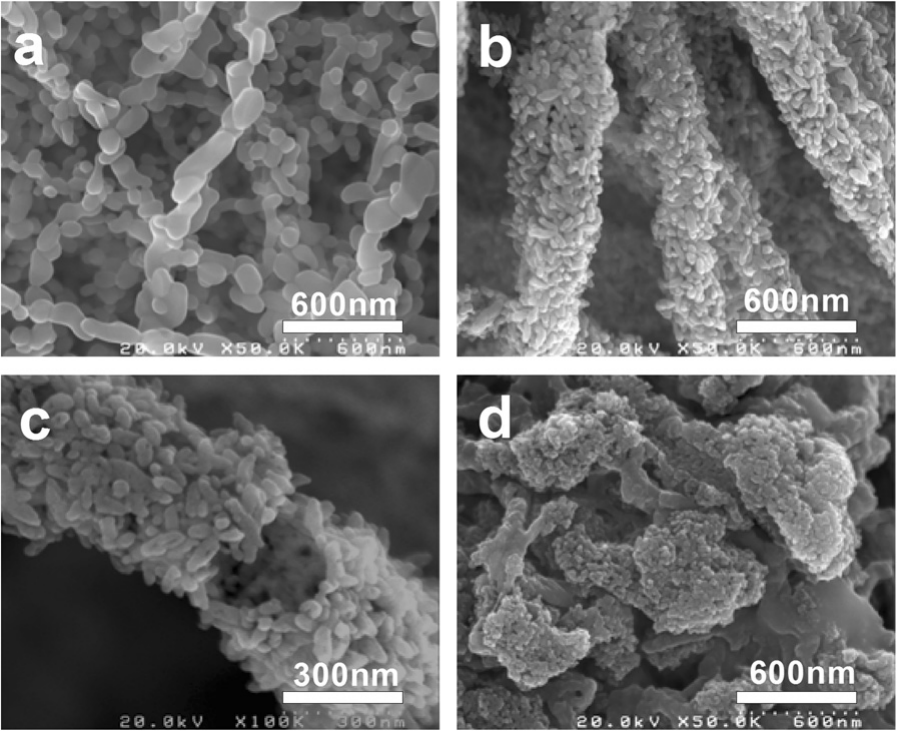
FE-SEM images of α-Fe_2_O_3_ with diverse shapes prepared at different ion concentrations. **(a)** a in Table 2; **(b, c)** b in Table 2; **(d)** d in Table 2.

α-Fe_2_O_3_ with diverse shapes was prepared from the same calcination process due to the different ion concentrations in hydrothermal reaction as shown in Table 2. In Figure 7, when the ion concentration is low, continuous fibers of interconnected nanorods were approached (Figure 7a), similar to the reported work [11]. When Fe^3+^ ion content increases to 21.1 μmol, novel hollow α-Fe_2_O_3_ nanofibers made of rice-like nanorods were prepared as shown in a representative high-resolution FE-SEM image (Figure 7b). In Figure 7c, hollow structure of α-Fe_2_O_3_ nanofibers can be clearly seen and abundant rice-like nanorods with the mean size of 60 ± 17 nm can be found. The hollow α-Fe_2_O_3_ nanofibers with hierarchical structure will find wide applications in photocatalyst, heavy metal ion detection, and lithium-ion battery due to the specific structure. However, tenfold increase in ion concentration resulted in only aggregates of α-Fe_2_O_3_ nanoparticles without fibrous morphology.

**Table 2:** **Ion concentration in the hydrothermal synthesis**

**α-Fe**_**2**_ **O**_**3**_	**FeCl**_**3**_ **·6H**_**2**_ **O (μmol)**	**FeCl**_**2**_ **·4H**_**2**_ **O (μmol)**	**NaOH (mol)**
a	2.11	1.05	2.12
b	21.1	10.5	21.2
d	211	105	212

### Magnetic response and absorption for dyes

**Figure 8:**
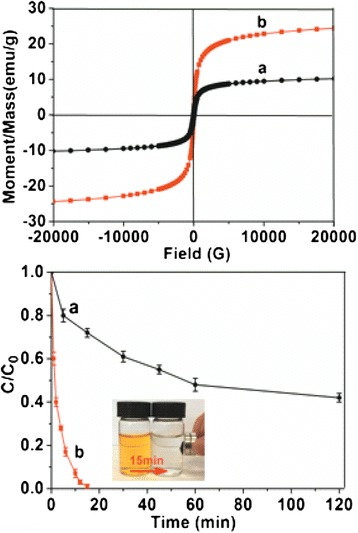
M-H curves and the adsorption capacity of MO of hollow α-Fe_2_O_3_ nanofibers. Magnetic hysteresis loops of α-Fe_2_O_3_ fibers measured at 300 K and the *C*/*C*_0_ versus time plots for adsorption of MO solution using **(a)** solid fibers and **(b)** hollow fibers made of rice-like nanorods; the inset shows the magnetic response of hollow α-Fe_2_O_3_ fibers made of rice-like nanorods after adsorption of MO for 15 min.

Nanostructured α-Fe_2_O_3_ displays weak ferromagnetic behavior at room temperature [28]. The M-H curves revealed in Figure 8 show a nonlinear and reversible behavior with a weak magnetic hysteresis loop. This was related to the fine crystallite sizes of α-Fe_2_O_3_ nanorods. The as-prepared hollow α-Fe_2_O_3_ nanofibers made of rice-like nanorods (Figure 7b) exhibited a robust saturation magnetization of 24.4 emu∙g^−1^, higher than 10.2 emu∙g^−1^ of solid fibers (Figure 7a) and that in the reported work [29], which should be ascribed to the hollow fiber structure and well-defined rice-like shape of α-Fe_2_O_3_.

As expected, hollow α-Fe_2_O_3_ nanofibers made of rice-like nanorods which combined the porous structure and the magnetic performance demonstrated efficient adsorption for organics and fast magnetic separation property. Methyl orange (MO; 2 × 10^−5^ M) was employed for typical organic pollutants in adsorption test. Figure 8 indicates that the adsorption capacity of MO of hollow α-Fe_2_O_3_ nanofibers was 93% for 10 min and could achieve almost complete adsorption of MO in 15 min (insert image of Figure 8) while solid α-Fe_2_O_3_ fibers revealed much slower adsorption rate. Moreover, hollow α-Fe_2_O_3_ nanofibers after adsorption could be separated facilely using an external magnet without any tedious separation process, which is of great importance for real applications.

**Figure 9:**
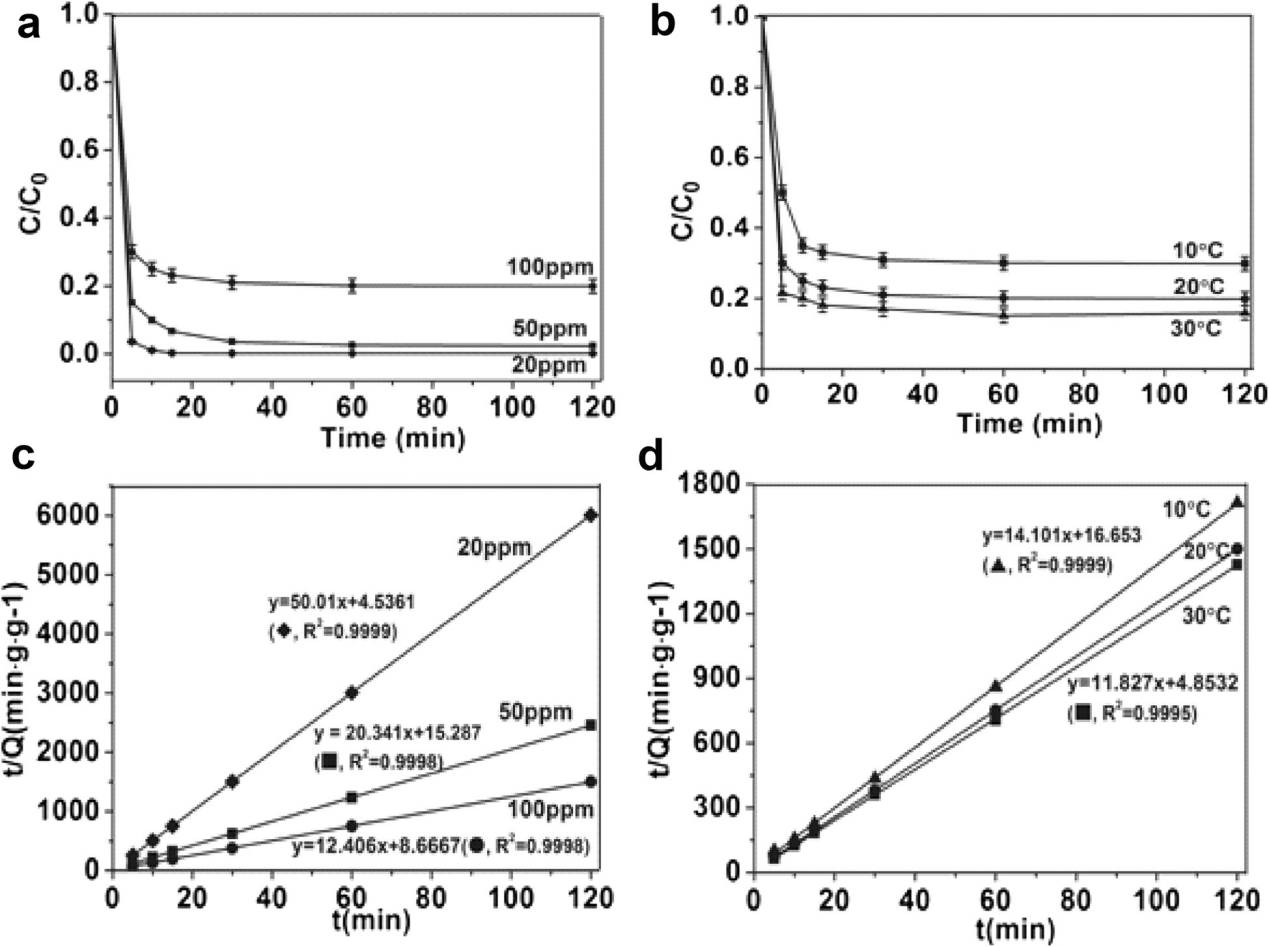
Adsorption curves and kinetics of hollow α-Fe_2_O_3_ fibers made of rice-like nanorods to MO. Different initial MO concentrations **(a, c)** and temperatures **(b, d)**.

Recently, environmental cleanup has been one of the most active areas in photocatalysis. An ideal photocatalyst should be stable, inexpensive, nontoxic, and, of course, highly photoactive [30]. Fe_2_O_3_ stands out with its nearly ideal bandgap of 2.2 eV and its high photochemical stability in aqueous solutions [31]. Yu et al*.* reported that degradation pathways of MO by using well-defined α-Fe_2_O_3_ nanocrystals involve both *N*-demethylation and the cleavage of conjugated chromophores [23]. To clear the adsorption kinetics of MO by using hollow α-Fe_2_O_3_ nanofibers made of rice-like nanorods, the effects of initial MO concentration and temperature were investigated systematically. As shown in Figure 9, at lower initial MO concentration (Figure 9a) or higher temperature (Figure 9b), better MO degradation performance was observed. Lagergren pseudo-second-order kinetic model was adopted to describe the adsorption process. *k*_2_ is the adsorption rate constant, *Q*_*e*_ is the equilibrium absorption capacity:

$$ \frac{t}{Q}=\frac{1}{k_2{Q}_e^2}+\frac{t}{Q_e} $$

Figure 9c shows that the adsorption curves to MO of hollow α-Fe_2_O_3_ nanofibers at different initial MO concentrations, which are based on the Lagergren pseudo-second-order kinetic equation. The change in initial MO concentration results in different molar ratio of hollow α-Fe_2_O_3_ nanofibers to MO, finally lead to varying *k*_2_. When initial MO concentration increased from 20 to 50 ppm, *k*_2_ dramatically reduced from 551.35 to 27.07 min^−1^. When the initial MO concentration is 100 ppm, *k*_2_ is 17.759 min^−1^, the equilibrium absorption capacity *Q*_*e*_ is 80.6 mg∙g^−1^. The increase of the initial MO concentration is negative to the adsorption rate, and this coincides with Figure 9a. The temperature of batch adsorption also affects the adsorption kinetics. As revealed in Figure 9d and Table 3, a 2.4-fold increase in *k*_2_ was achieved with the increase of experimental temperature from 10°C to 30°C.

**Table 3:** **Adsorption rate constant** ***k***_**2**_ **at different initial MO concentration and temperature**

**Adsorption condition**	**Initial MO concentration/ppm (at 20°C)**			**Temperature/°C (at 100 ppm)**		
	**20**	**50**	**100**	**10**	**20**	**30**
*k*_2_	551.35	27.07	17.76	11.94	17.76	28.82

## Conclusions

Novel hollow α-Fe_2_O_3_ nanofibers made of rice-like nanorods were successfully synthesized via a simple hydrothermal reaction on PVA nanofiber template followed by calcination. The crystallographic structure and the morphology of the as-prepared α-Fe_2_O_3_ nanofibers were conformed by XRD, EDS, and FE-SEM. Moreover, hollow α-Fe_2_O_3_ fiber assembles exhibited magnetic responsive performance, as well as efficient adsorption for methyl orange in water which follows Lagergren pseudo-second-order kinetics. This work provided a versatile strategy for further design and development of functional nanofiber-nanoparticle composites towards various applications.
